# Evaluating Stable Chronic Obstructive Pulmonary Disease by Ultrasound

**DOI:** 10.1155/2019/5361620

**Published:** 2019-09-15

**Authors:** Togay Evrin, Semih Korkut, Leyla Ozturk Sonmez, Lukasz Szarpak, Burak Katipoglu, Jacek Smereka, Ramazan Guven, Evrim Eylem Akpinar

**Affiliations:** ^1^Department of Emergency Medicine, Ufuk University Medical Faculty, Dr. Ridvan Ege Education and Research Hospital, Ankara, Turkey; ^2^Department of Emergency Medicine, University of Health Sciences, Kartal Dr. Lütfi Kırdar Training and Research Hospital, Istanbul, Turkey; ^3^Department of Physiology, Selcuk University Faculty of Medicine, Konya, Turkey; ^4^Department of Emergency Medicine, Necmettin Erbakan University, Meram Faculty of Medicine, Konya, Turkey; ^5^Lazarski University, Medical Faculty, Warsaw, Poland; ^6^Department of Emergency Medical Services, Wroclaw Medical University, Wroclaw, Poland; ^7^Department of Emergency Medicine, University of Health Sciences, Kanuni Sultan Süleyman Training and Research Hospital, Istanbul, Turkey; ^8^Department of Chest Diseases, Ufuk University Medical Faculty, Dr Ridvan Ege Education and Research Hospital, Ankara, Turkey

## Abstract

**Background and Aim:**

The purpose of the study was to evaluate the relationship between COPD severity and the diaphragmatic function measured by point-of-care US in patients with stable COPD.

**Method:**

A total of 61 patients with COPD and 40 healthy subjects who had been admitted to Ufuk University Hospital between December 2018 and May 2019 were enrolled. Point-of-care US was performed, and lung silhouette and anterior, right, and left hemidiaphragm method in M-mode were used to evaluate the diaphragm.

**Results:**

The point-of-care US measurements, lung silhouette method right (Lung Sil R), lung silhouette method left (Lung Sil L), right hemidiaphragm US method in B-mode (Ant B-Mode R), and right hemidiaphragm US method in M-mode (Ant M-Mode R), were significantly different among groups (*P* < 0.001 for each). FEV1 was strongly correlated with Lung Sil R, Lung Sil L, Ant B-Mode R, and Ant M-Mode R (*r* = 0.963, *P* < 0.001; *r* = 0.956, *P* < 0.001; *r* = 0.953, *P* < 0.001; and *r* = 0.917, and *P* < 0.001, respectively). Negative correlations were detected between the number of exacerbations per year and Lung Sil R and the number of exacerbations per year and Ant M-Mode R (*r* = −0.599, *P* < 0.001 and *r* = −0.587, and *P* < 0.001, respectively).

**Conclusion:**

In this study, FEV1 and annual number of exacerbations turned out to be strongly correlated US findings. The use of US in COPD patients could help to support clinical decision, but further clinical studies are necessary to confirm those findings.

## 1. Introduction

Chronic obstructive pulmonary disease (COPD) is a heterogeneous and progressive disease characterized by restricted airflow. It is associated with high morbidity and mortality and increasing social and economic burdens worldwide [1, 2]. Previous studies have shown that the forced expiratory volume in 1 s (FEV1) is not an adequate measure to determine the severity of COPD [3]. For this reason, the 2011 update of the Global Initiative for Chronic Obstructive Lung Disease (GOLD) criteria proposed an assessment based on symptoms and inflammation to determine the disease severity, manage the treatment, and estimate the prognosis of COPD [3]. In contrast, the 2017 update suggested that spirometry be used exclusively to diagnose COPD and that FEV1 be removed from the evaluation because this value may cause confusion when defining disease groups [4]. In addition to airway obstruction, myopathy due to systemic inflammation and mechanical effects due to hyperinflation can lead to diaphragmatic dysfunction in patients with COPD. Oxidative stress, muscle loss, reduced protein production, and increased apoptosis also contribute to diaphragmatic dysfunction. The mass, thickness, and area of the diaphragm vary in patients with COPD [5, 6]. The diaphragm is the most important respiratory muscle involved in maintaining ventilation. Although the diaphragm moves in a range of 1-2 cm during resting breathing, its movement during forced breathing reaches 7–11 cm [7].

As no suitable tests are available to determine the function of the diaphragm, diaphragmatic dysfunction is not generally recognized; however, the need for an evaluation is significant for both inpatients and outpatients, especially during emergencies [8]. There are many methods to evaluate diaphragmatic function, of which transdiaphragmatic pressure measurement is the gold standard for diagnosis [9, 10].

The techniques traditionally used to diagnose diaphragmatic weakness or paralysis are invasive, expose patients to radiation, or require them to leave the room (electromyography and fluoroscopy). In addition, they may be time-consuming, indirect, and uncomfortable (transdiaphragmatic pressure measurement and plethysmography) or complex and expensive (dynamic ecoplanar magnetic resonance imaging) [6].

M- and B-mode ultrasonography (US) was first used by Haber et al. in 1975 to evaluate diaphragm movement [11]. Subsequently, despite the many definitions for diaphragmatic movement and tidal volume, the value of bedside US for evaluating diaphragmatic paralysis in intensive care was confirmed by Dorffner et al. in 1998, with a 100% sensitivity [12]. Quantitative US, which is applied to assess severe diaphragmatic dysfunction on the basis of transdiaphragmatic pressure measurements, was described by Lerolle et al. in 2009 [13].

The use of US for structural and functional evaluations of the diaphragm is increasing. It has been reported that diaphragmatic thickness fraction measurements are suitable for determining lung hyperinflation in patients with COPD [2]. For this reason, diaphragm examination by point-of-care US may be helpful for evaluating the disease status and outcomes in COPD patients [2, 14].

The purpose of the present study was to evaluate the relationship between COPD severity and the diaphragmatic function measured by point-of-care US in patients with stable COPD.

## 2. Materials and Methods

### 2.1. Study Design and Setting

This observational case-control study was performed at a large tertiary referral academic institution after receiving the institutional review board approval. All patients provided their verbal consent and signed a written consent form. The diagnosis of COPD was based on medical history, clinical examinations, and pulmonary function tests (PFTs), and all diagnoses were made in accordance with the GOLD criteria [4]. The patients were asked about their history of tobacco smoking, biomass smoke exposure history, annual number of exacerbations, presence of concomitant conditions, and disease duration. The symptom scores were calculated with the use of the modified Medical Research Council Dyspnea Scale [15].

The study involved patients aged >40 years who had a post-bronchodilator FEV1/forced vital capacity ratio <70% on PFTs (Vmax® Encore PFT System; CareFusion, Yorba Linda, CA, USA) performed by the same trained operator in accordance with the American Thoracic Society standards. A total of 61 patients with COPD and 40 healthy subjects who had been admitted to Ufuk University Hospital between December 2018 and May 2019 were enrolled. Patients with malignancies, neuromuscular conditions, cerebrovascular diseases, unilateral or bilateral pleural effusion, pneumothorax, atelectasis, pneumonia, interstitial lung diseases, recent surgical operations, COPD exacerbations within the previous 3 months, and refusal to participate in the study were excluded. Comorbidities, including cardiac insufficiency, hypertension, renal insufficiency, and diabetes mellitus, were queried and recorded.

### 2.2. Measurements

US was performed with a Terason Usmart 3200T ultrasound system (77 Terrace Hall Avenue Burlington, MA 01803 United States) and a 3.5 MHz curved probe.

#### 2.2.1. Lung Silhouette Method (Lung Sil Right and Lung Sil Left)

**Figure 1:**
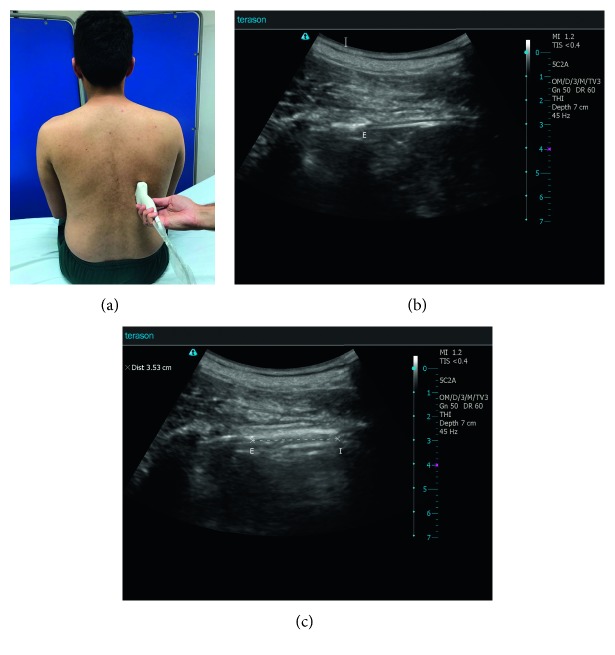
(a) Sonographic measurement of the upward and downward movements of the lung silhouette, here on the right side (lung silhouette method). The patient is sitting; the transducer is placed at the lowest point of the lung silhouette in the scapular line. While the patient breathes as deeply as possible, a video sequence is performed. Afterward, the distance between maximal inspiration and maximal expiration can be measured. (b) Sonographic measurement of the upward and downward movements of the lung silhouette, here on the right side. E marks the lowest point of the lung silhouette at maximal end expiration. (c) Sonographic measurement of the upward and downward movements of the lung silhouette—here on the right side. E marks the lowest point of the lung silhouette at maximal end expiration, and I marks the lowest point at maximal inspiration. In this example, the distance between E and I is 35.3 mm.

The upward and downward movements of the lung silhouette in the scapular line were measured. All participants were evaluated in a sitting position. The transducer was placed at the lowest part of the lung silhouette in the scapular line. The probe orientation should be longitudinal scan. The patient was instructed to exhale as deeply as possible to the residual volume and then to inhale deeply to the total lung capacity. This manoeuver was filmed, and the distance between the highest and lowest points of the lung silhouette was measured 3 times and the mean value was calculated. This manoeuver was performed on the right and left sides [16–20] (Figures 1(a)–1(c)).

#### 2.2.2. Right Hemidiaphragm US Method in B-Mode (Ant B-Mode Right)

**Figure 2:**
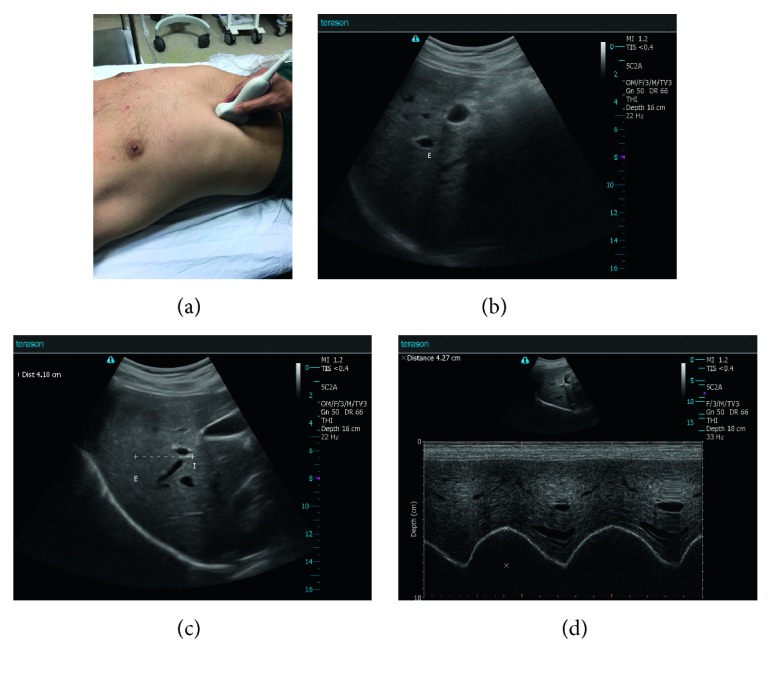
(a) The convex probe is positioned on the abdomen to examine the right diaphragmatic dome. The upward and downward movements of the right diaphragmatic dome were measured from the anterior position. The probe orientation should be longitudinal scan (right hemidiaphragm US method in B-mode and M-mode). (b and c) B-mode ultrasound evaluation of the craniocaudal displacement of the left branch of the portal vein in a patient with COPD. The position of the vessel was marked by the calliper during forced expiration and inspiration manoeuvres. The line shows displacement of the left branch of the portal vein during maximal inspiratory and expiratory breathing in the sagittal plane. The craniocaudal displacement of this branch was registered in millimetres. E marks at maximal end expiration, and I marks the lowest point at maximal inspiration. The distance between E and I is 41 mm (Ant B-Mode R). (d). M-mode scan of the right hemidiaphragmatic dome at maximal inspiration The first calliper was placed at the foot of the inspiration slope on the diaphragm echoic line and the second one at the apex of this slope for the deep breathing measurements (Ant M-Mode R: 42.7 mm).

The upward and downward movements of the right diaphragmatic dome were measured from the anterior position. All participants were evaluated by US in a completely supine position. The transducer was placed in the area between the anterior axillary line and the midclavicular line, with the liver as a US window directed toward the diaphragmatic dome. The probe orientation should be longitudinal scan. The participant was instructed to exhale as deeply as possible to the residual volume and then to inhale deeply to the total lung capacity. This manoeuver was filmed, and the distance between the highest and lowest points of the right hemidiaphragmatic dome was measured. This method was performed only on the right hemidiaphragmatic side because of the known difficulties that accompany left side measurements with the spleen and stomach as the US window (Figures 2(a)–2(c)) [16–20].

#### 2.2.3. Right and Left Hemidiaphragms US Method in M-Mode (Ant B-Mode Right and Ant B-Mode Left)

The probe was placed between the midclavicular and anterior axillary lines into the subcostal area and was directed medially, cranially, and dorsally, so that the US beam was perpendicular to the posterior third of the right and left hemidiaphragms. The probe orientation should be longitudinal scan. Diaphragmatic movements were recorded in M-mode. This manoeuver was started at the end of normal expiration, and the volunteers and COPD patients were asked to inhale as deeply as possible. The subcostal or low intercostal probe position was chosen between the anterior and mid axillary lines to obtain the best image of the left hemidiaphragmatic dome. Motion was recorded during the same respiratory manoeuvers as for the right hemidiaphragm. The inspiratory amplitudes (excursions) of the diaphragm were measured by M-mode US. The first calliper was placed at the foot of the inspiration slope on the diaphragm echoic line and the second one at the apex of this slope for the deep breathing measurements (Figure 2(d)) [20].

All ultrasonographic measurements (lung silhouette method, right hemidiaphragm US method in B-mode, and right and left hemidiaphragms US method in M-mode) were made by the same emergency medicine specialist certified in lung and diaphragmatic US (POCUS), who was blinded to the clinical characteristics and pulmonary function status of each patient. Several respiratory cycles were recorded, and the measurements from at least three different cycles were averaged for each US method.

### 2.3. Statistical Analysis

The data were statistically analysed with the SPSS 25.0 software (IBM Corp., Armonk, NY, USA). The categorical measurements were reported as numbers and percentages, and the Shapiro–Wilk test was performed to determine the normality of the continuous variable distributions, the results of which are presented as medians (quartiles). The Kruskal–Wallis test served to compare nonnormally distributed variables. Differences in the continuous variables among the four groups were considered significant at *P* < 0.05/6. The chi-squared test was applied to compare the categorical variables, and the Monte Carlo simulation test was used for the significance level of scores <5. Spearman's correlation analysis allowed to determine the correlations among the US measurements: lung silhouette right (Lung Sil R), lung silhouette left (Lung Sil L), anterior B-mode right (Ant B-Mode R), anterior M-mode right (Ant M-Mode R), and anterior M-mode left (Ant M-Mode L). The level of *P* < 0.05 was considered significant.

## 3. Results

**Figure 3:**
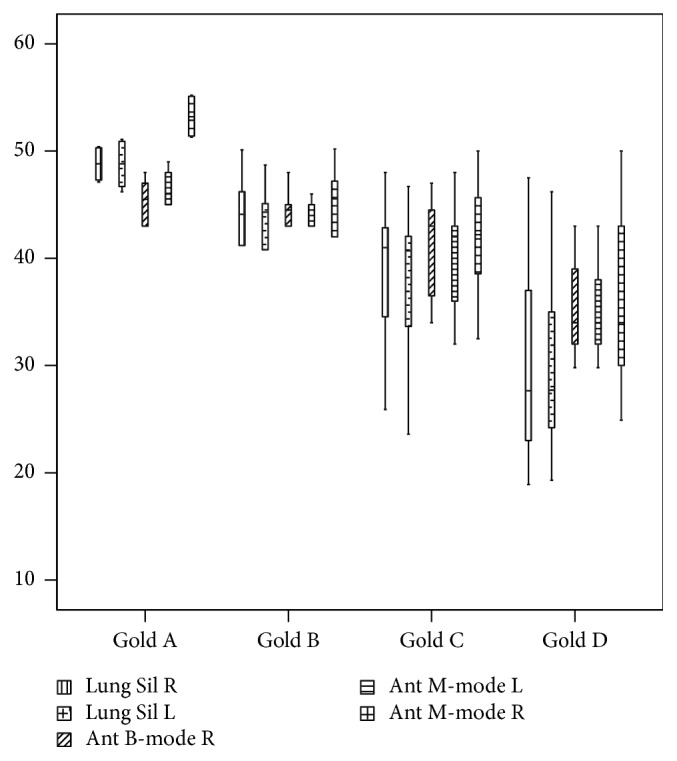
Box and whisker plot. Lung Sil R (mm), Lung Sil L (mm), Ant M-mode R (mm), Ant M-Mode L (mm), and Ant B-Mode R (mm) values in patients with COPD in accordance with GOLD classification in 95% confidence interval.

A total of 85 patients with COPD met the inclusion criteria of this study. Of these, 21 were excluded because they had suffered from COPD exacerbations within the past 3 months, and 3 were excluded because they could not undergo bedside US owing to patient or technical limitations. Therefore, 61 patients with COPD who did not meet the exclusion criteria were included in the study; 80.3% (*n* = 49) were males and 19.7% (*n* = 12) were females. The median age of the participants was 70.0 (interquartile range (IQR), 64.0–78.5) years. The demographic characteristics of control group and GOLD A, GOLD B, GOLD C, and GOLD D groups are shown in Table 1, with sex, age, and annual number of seizures different among the groups (*P* < 0.001 for each). The number of annual exacerbations was the highest in GOLD D group and the median (IQR) annual number of exacerbations in this group equalled 2.0 (1.0). Five point-of-care US measurements, Lung Sil R, Lung Sil L, Ant B-Mode R, Ant M-Mode R, and Ant M-Mode L, were significantly different among the patient groups (*P* < 0.001 for each) (Table 2). The lowest and highest median values of these US measurements were detected among patients in GOLD D group and those in GOLD A group, respectively (lowest vs. highest: 27.6 vs. 48.8 mm, 27.7 vs. 48.8 mm, 34.0 vs. 53.2 mm, 34 vs. 45 mm, and 34 vs. 46 mm, respectively) (Figure 3).

**Figure 4:**
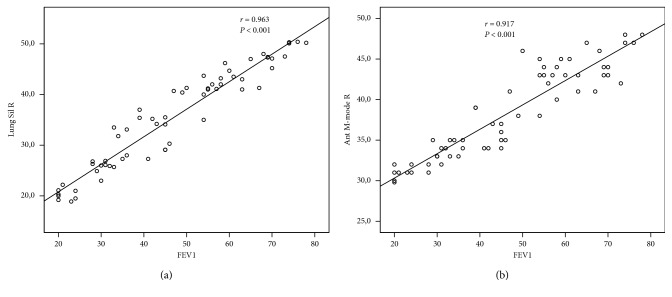
Correlation between (a) Lung Sil R (mm) and FEV1 (%) in COPD patients and (b) Ant M-Mode R (mm) and FEV1 (%) in COPD Patients.

The correlations between the US findings and FEV1 and their significance levels are shown in Table 3. FEV1 was strongly correlated with Lung Sil R, Lung Sil L, Ant B-Mode R, Ant M-Mode R, and Ant M-Mode L L (*r* = 0.963, *P* < 0.001; *r* = 0.956, *P* < 0.001; *r* = 0.953, *P* < 0.001; and *r* = 0.917, *P* < 0.001; *r* = 0.947, *P* < 0.001, respectively) (Figures 4(a) and 4(b)).

**Figure 5:**
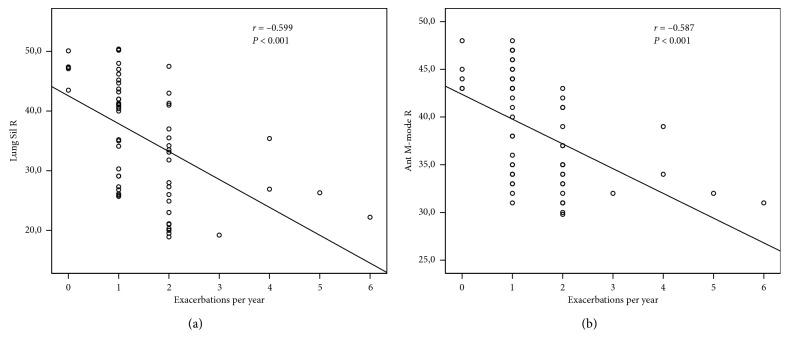
Correlation between (a) Lung Sil R (mm) and number of exacerbations per year and (b) Ant M-Mode R (mm) and number of exacerbations per year.

Significant negative correlations were detected between the number of exacerbations per year Lung Sil R and Ant M-Mode R (*r* = −0.599, *P* < 0.001; *r* = −0.587, *P* < 0.001, respectively) (Figures 5(a) and 5(b)).

The control group consisted of 40 healthy volunteers; 85.0% (*n* = 34) were males and 15% (*n* = 6) were females, with a median age of 67.5 (IQR, 63.0–73.0) years. The median US (IQR) values were 66.0 mm (4.5) for Lung Sil R, 66.1 mm (4.0) for Lung Sil L, 69.0 mm (3.8) for Ant B-Mode R, and 7.1 cm (0.5) for Ant M-Mode R. The statistical significance of the US finding differences between the control and COPD groups is shown in Table 2.

For the assessment of the US operator's skills across the study period, the correlation between FEV1-US findings (e.g., Lung Sil R) of the first 10 patients and the FEV1-US findings (e.g., Lung Sil R) of the last 10 patients was evaluated (*r* = 0.936, *P* < 0.001). It was observed that the correlation between FEV1-Lung Sil R of the first and the last 10 patients (*r* = 0.912, *P* < 0.001) was strong.

## 4. Discussion

This is the first study conducted among patients with COPD who were graded in accordance with the updated GOLD classification. Some research concerning lung US followed the former GOLD classification. In many studies, diaphragm replacement was measured with the use of FEV1, and the lung silhouette and anterior US B-mode measurements were correlated with the M-mode measurements [19, 21]. Therefore, new studies need to be performed on the new GOLD classification with respect to US. In our study, patients were classified with the new GOLD classification, and both the right and left US measurements were compared; the diaphragmatic measurements using the lung silhouette method were strongly correlated with the FEV1, anterior method B-mode, and M-mode measurements.

The lung silhouette method is a technologic-device-supported model of the lung percussion in which diaphragmatic dysfunction is evaluated by the up and down movements of the right and left hemidiaphragms over the scapular line. The method is a version of the former diaphragm model.

Previous studies showed that the results of this method were strongly correlated with those of the anterior axillary method and that the method was easily performed in all patients, including obese ones, feasibly applied together with US, and suitable for evaluating both hemidiaphragms [19, 22]. In our study, no significant differences were detected between the techniques in terms of measuring diaphragmatic dysfunction on both sides using the anterior method, and the correlations between the two techniques were strong. Although no significant differences were detected between the right and left evaluations when using either method, evaluation based on the right anterior method measurements may be more useful, especially in patients who cannot assume the interscapular image position during point-of-care US. Creating a window and capturing the appropriate image may be more difficult on the left side because of gastric gas [23], so the right side is preferred for imaging convenience.

One of the most important findings of the present study was the strong correlation detected between the lung silhouette images taken from the interscapular line and images taken with the anterior method and FEV1. A similar correlation was found in a previous study; however, the correlation coefficient was not 100% [1]. The authors of that study attributed their results to different diaphragmatic effects among patients with different types of emphysema. Some studies report that diaphragmatic dysfunction may be different in basal-predominant and apical-predominant emphysema [24]. As the lung silhouette method and M-mode measurements have been correlated with FEV1 [25, 26], the operator can use the anterior axillary method in patients who are unable to sit and the lung silhouette method in those who cannot reach. Depending on the condition of the patient or the comfort of the doctor, both methods may be preferred, particularly for emergency services, when the patient is difficult to position.

Another important finding of the present study was that as the number of exacerbations per year increased in a patient, the measurements made with the lung silhouette and anterior axillary methods showed negative correlations. The US measurements of the patients were not taken during an exacerbation. However, the role of US diaphragmatic measurements in predicting the number of exacerbations per year should be clarified via more comprehensive studies conducted on the basis of our study results. This may help emergency medicine physicians in deciding on discharge vs. hospitalization.

Patients with stable COPD were included in the present study. For this reason, the use of data from emergency services may be confusing; however, patients who are admitted with respiratory distress to emergency services are relieved by various treatments. The results of this study may help in making the decision on hospitalization vs. discharge after the recovery of the patient's condition. In addition, this is a pioneer study in terms of its implementation in emergency services. Further research is needed to determine the predictive power of a diaphragmatic functional evaluation by US. If the severity of the exacerbations can be determined with US in the most comfortable position, US may play a role in the decision on early intubation, intensive care follow-up, or hospitalization. A multicentre study in a larger group of patients with exacerbations is needed to support the findings of our study.

The study had some limitations. First, the number of patients with COPD was limited, and the study was conducted at a single centre. Thus, the population might not be representative of all patients with COPD. Second, the diaphragmatic measurements were performed by only one emergency specialist. This may have caused an underestimation of the results based on interobserver variations. The inclusion of patients with stable COPD only was another limitation, because the results are not applicable to COPD patients during an exacerbation period, and this fact limits the therapeutic decisions to be made based on US in exacerbated patients. Since an adequate measurement of diaphragmatic thickness fraction is difficult in daily practice, we did not use its parameters in this study.

## 5. Conclusion

In this study, FEV1 and annual number of exacerbations turned out strongly correlated US findings. The use of US in COPD patients could help to support clinical decision, but further clinical studies are necessary to confirm those findings.

## Figures and Tables

**Table 1:** Demographic characteristics and significance levels in control group and COPD patients according to their stages.

	Control group (*n* = 40)	GOLD A (*n* = 6)	GOLD B (*n* = 6)	GOLD C (*n* = 15)	GOLD D (*n* = 34)	*P* value
Male, *n* (%)	34 (85.0)	1 (16.7)	3 (50.0)	13 (86.7)	32 (94.1)	<0.001
Age, median (IQR)	67.5 (10.0)	58.0 (7.0)	78.5 (15.0)	65.0 (16.0)	71.0 (13.0)	<0.001
Number of exacerbations in the previous year, median (IQR)	—	0.50 (1.0)	1.0 (1.0)	1.0 (0.0)	2.0 (1.0)	<0.001
Smoking, *n* (%)	23 (57.5)	6 (100.0)	6 (100.0)	15 (100.0)	31 (91.2)	0.836
BMI, median (IQR)	27.1 (3.8)	28.2 (5.1)	25.9 (6.5)	26.5 (7.5)	24.5 (4.0)	0.095
CHD, *n* (%)	1 (2.5)	0 (0.0)	3 (50.0)	2 (13.3)	11 (32.4)	0.115
CHF, *n* (%)	1 (2.5)	3 (50.0)	1 (16.7)	2 (13.3)	4 (11.8)	0.115
DM, *n* (%)	5 (12.5)	0 (0.0)	1 (16.7)	6 (40.0)	9 (26.5)	0.279
HT, *n* (%)	10 (25.0)	6 (100.0)	1 (16.7)	10 (66.7)	20 (58.8)	0.033

Data are expressed as mean ± standard deviation for normally distributed data and percentage for categorical variables. COPD: chronic obstructive pulmonary disease; GOLD: Global Initiative for Chronic Obstructive Lung Disease; IQR: interquartile range; BMI: body mass index; CHD: coronary heart disease; CHF: congestive heart failure; DM: diabetes mellitus; HT: hypertension.

**Table 2:** Pulmonary function test values and significance levels with ultrasonographic findings according to the COPD stage and control group.

	Control group (*n* = 40)	GOLD A (*n* = 6)	GOLD B (*n* = 6)	GOLD C (*n* = 15)	GOLD D (*n* = 34)	*P* value
Lung Sil R (mm), median (IQR)	66.0 (4.5)	48.8 (3.1)	44.1 (9.5)	41.0 (9.6)	27.6 (15.1)	<0.001
Lung Sil L (mm), median (IQR)	66.1 (4.0)	48.8 (4.4)	44.3 (8.9)	40.7 (9.1)	27.7 (12.1)	<0.001
Ant B-Mode R (mm), median (IQR)	71 (5.0)	53.2 (3.8)	45.5 (8.2)	42.2 (8.5)	34.0 (13.0)	<0.001
Ant M-mode R (mm), median (IQR)	69.0 (3.8)	45 (4.0)	44 (5.0)	43 (9.0)	34 (7.0)	<0.001
Ant M-mode L (mm), median (IQR)	70 (6.0)	46 (3.0)	45 (5.0)	42 (7.0)	34 (6.0)	<0.001
FVC (%), median (IQR)	98.5 (20)	92.5 (7)	72.0 (18.0)	67.0 (17.0)	55.0 (29.0)	<0.001
FEV1 (%), median (IQR)	94.0 (19)	72.0 (8.0)	59.5 (14.0)	54.0 (18.0)	34.5 (19.0)	<0.001
FEV1/FVC (%), median (IQR)	91.0 (9)	66.5 (2.0)	63. (6.0)	63.0 (13.0)	47.5 (18.0)	<0.001

COPD: chronic obstructive pulmonary disease; GOLD: Global Initiative for Chronic Obstructive Lung Disease; IQR: interquartile range; FVC: forced vital capacity; FEV1: forced expiratory volume in 1 s.

**Table 3:** Correlation between FEV1 and ultrasonographic findings.

Variables	Control (*n* = 40)		COPD (*n* = 61)	
	Correlation coefficient	*P* value	Correlation coefficient	*P* value
Lung Sil R	0.522	**0.001**	0.963	**<0.001**
Lung Sil L	0.535	**<0.001**	0.956	**<0.001**
Ant B-Mode R	0.599	**<0.001**	0.953	**<0.001**
Ant M-Mode R	0.682	**<0.001**	0.917	**<0.001**
Ant M-Mode L	0.747	**<0.001**	0.947	**<0.001**

FEV1: forced expiratory volume in 1 s.

## Data Availability

The US findings data used to support the findings of this study have been deposited in the Togay Evrin's repository.

## References

[1] Bastian A., Scheibe N., Sosnowski N., Pinkhasik A., Vonderbank S. (2015). Sonographic evaluation of diaphragmatic dysfunction in COPD patients. *International Journal of Chronic Obstructive Pulmonary Disease*.

[2] Eryüksel E., Cimşit C., Bekir M., Cimsit Ç., Karakurt S. (2017). Diaphragmatic thickness fraction in subjects at high-risk for COPD exacerbations. *Respiratory Care*.

[3] Global Initiative for Chronic Obstructive Lung Disease (GOLD) (2011). *Global Strategy for the Diagnosis, Management and Prevention of COPD*.

[4] The Global Initiative for Chronic Obstructive Lung Disease (GOLD) (January 2017). Global strategy for the diagnosis, management, and prevention of chronic obstructive pulmonary disease.

[5] Ottenheijm C. A. C., Heunks L. M. A., Sieck G. C. (2005). Diaphragm dysfunction in chronic obstructive pulmonary disease. *American Journal of Respiratory and Critical Care Medicine*.

[6] Smargiassi A., Inchingolo R., Tagliaboschi L., Di Marco Berardino A., Valente S., Corbo G. M. (2014). Ultrasonographic assessment of the diaphragm in chronic obstructive pulmonary disease patients: relationships with pulmonary function and the influence of body composition—a pilot study. *Respiration*.

[7] Yamaguti W. P. d. S., Paulin E., Shibao S., Kodaira S., Chammas M. C., Carvalho C. R. F. (2007). Avaliação ultra-sonográfica da mobilidade do diafragma em diferentes posturas em sujeitos saudáveis. *Jornal Brasileiro de Pneumologia*.

[8] Wilcox P. G., Pardy R. L. (1989). Diaphragmatic weakness and paralysis. *Lung*.

[9] McCool F. D., Tzelepis G. E. (2012). Dysfunction of the diaphragm. *New England Journal of Medicine*.

[10] Green M., Road J., Sieck  G. C., Similowski T. (2002). ATS/ERS statement on respiratory muscle testing. *American Journal of Respiratory and Critical Care Medicine*.

[11] Haber K., Asher W. M., Freimanis A. K. (1975). Echographic evaluation of diaphragmatic motion in intra-abdominal diseases. *Radiology*.

[12] Dorffner R., Eibenberger K., Youssefzadeh S. (1998). Wertigkeit der sonographie auf der intensivstation zur diagnostik von zwerchfellparesen. *RöFo—Fortschritte auf dem Gebiet der Röntgenstrahlen und der bildgebenden Verfahren*.

[13] Lerolle N., Guérot E., Dimassi S. (2009). Ultrasonographic diagnostic criterion for severe diaphragmatic dysfunction after cardiac surgery. *Chest*.

[14] Okura K., Kawagoshi A., Iwakura M. (2017). Contractile capability of the diaphragm assessed by ultrasonography predicts nocturnal oxygen saturation in COPD. *Respirology*.

[15] Gerlach Y., Williams M. T., Coates A. M. (2013). Weighing up the evidence-a systematic review of measures used for the sensation of breathlessness in obesity. *International Journal of Obesity*.

[16] Ueki J., De Bruin P. F., Pride N. B. (1995). In vivo assessment of diaphragm contraction by ultrasound in normal subjects. *Thorax*.

[17] Cohn D., Benditt J. O., Eveloff S., McCool F. D. (1997). Diaphragm thickening during inspiration. *Journal of Applied Physiology*.

[18] Toledo N. S. G., Kodaira S. K., Massarollo P. C. B., Pereira O. I., Mies S. (2003). Right hemidiaphragmatic mobility: assessment with US measurement of craniocaudal displacement of left branches of portal vein. *Radiology*.

[19] Boussuges A., Gole Y., Blanc P. (2009). Diaphragmatic motion studied by M-mode ultrasonography. *Chest*.

[20] Houston J. G., Morris A. D., Howie C. A., Reid J. L., McMillan N. (1992). Technical report: quantitative assessment of diaphragmatic movement—a reproducible method using ultrasound. *Clinical Radiology*.

[21] Testa A., Soldati G., Giannuzzi R., Berardi S., Portale G., Gentiloni Silveri N. (2011). Ultrasound M-mode assessment of diaphragmatic kinetics by anterior transverse scanning in healthy subjects. *Ultrasound in Medicine & Biology*.

[22] Topeli A., Laghi F., Tobin M. J. (2001). The voluntary drive to breathe is not decreased in hypercapnic patients with severe COPD. *European Respiratory Journal*.

[23] Goligher E. C., Laghi F., Detsky M. E. (2015). Measuring diaphragm thickness with ultrasound in mechanically ventilated patients: feasibility, reproducibility and validity. *Intensive Care Medicine*.

[24] Iwasawa T., Takahashi H., Ogura T. (2011). Influence of the distribution of emphysema on diaphragmatic motion in patients with chronic obstructive pulmonary disease. *Japanese Journal of Radiology*.

[25] Pellegrino R., Viegi G., Brusasco V. (2005). Interpretative strategies for lung function tests. *European Respiratory Journal*.

[26] Zanforlin A., Smargiassi A., Inchingolo R., di Marco Berardino A., Valente S., Ramazzina E. (2014). Ultrasound analysis of diaphragm kinetics and the diagnosis of airway obstruction: the role of the M-mode index of obstruction1. *Ultrasound in Medicine & Biology*.

